# Solitary metastasis of clear cell renal cell carcinoma in sternum diagnosed unexpectedly during cardiac surgery – A rare but potentially fatal trap

**DOI:** 10.1016/j.eucr.2021.101730

**Published:** 2021-05-28

**Authors:** Martin Dergel, Michal Balik, Jaroslav Pacovsky, Martin Vobornik, Jiri Mandak, Jan Laco

**Affiliations:** a Department of Cardiac Surgery, Charles University Faculty of Medicine in Hradec Kralove and University Hospital Hradec Kralove, Czech Republic; b Department of Urology, Charles University Faculty of Medicine in Hradec Kralove and University Hospital Hradec Kralove, Czech Republic; c The Fingerland Department of Pathology, Charles University Faculty of Medicine in Hradec Kralove and University Hospital Hradec Kralove, Czech Republic

**Keywords:** Clear cell renal cell carcinoma, Solitary metastasis, Sternum, Fatal complication, Cardio surgery, Section headings: oncology, RCC, clear cell renal cell carcinoma, CT, computed tomography

## Abstract

We present a very rare case of fatal complication during the cardiac surgery caused by unrecognized solitary metastasis of clear cell renal cell carcinoma in the sternum.

## Introduction

Renal carcinomas represent approximately 2–3% of all cancers in men. The incidence is increasing by 2% annually. In the *Czech* Republic, the age-standardized incidence rate was 14.7 per 100,000 people in 2018, which was the fourth highest worldwide (after Belarus, Latvia, and Lithuania).^1^ Approximately 30% of patients have distant metastases at the time of diagnosis. Another 14–29% of patients develop metastases during the follow-up period - even many years after the diagnosis.^2^

We present a very rare case of serious complication during cardiac surgery due to as yet unrecognized metastasis of clear cell renal cell carcinoma (RCC) to the sternum.

### Case

**Fig. 1:**
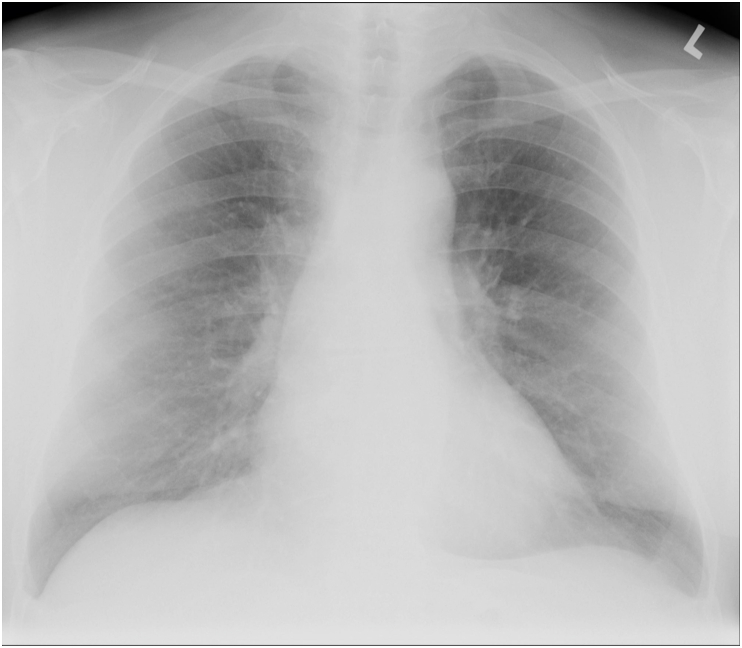
Preoperative chest x-ray – no visible metastasis.

A 66-year-old male with serious stenosis of the bicuspid aortic valve and significant mitral insufficiency was indicated for aortic valve replacement and mitral valve plasty. Besides “common” comorbidities (arterial hypertension, dyslipidaemia, etc.) the patient had undergone left nephrectomy for RCC (pT1a cN0 cM0; WHO grade III) five years before the planned cardiac surgery. No recurrence of RCC during the regular high-risk follow-up regimen was observed. The last follow up visit took place 6 months before the cardiac surgery. There was no pathologic finding on preoperative chest x-ray (Fig. 1). We did not find any palpable pathologic mass during preoperative chest examination. The patient's body mass index was 34.0, and ECOG/WHO performance status was 2.

The cardiac surgery was performed by surgeon with experience of hundreds difficult cases. At the beginning of the procedure, immediately after the skin incision, serious bleeding from the soft tissue occurred. All attempts to stop, or at least reduce, massive bleeding were ineffective. Therefore, the patient was connected to the extracorporeal circulation via the right femoral artery and vein. A sample from atypical subcutaneous “fatty” tissue was sent for frozen section which showed the metastasis of RCC. Resection of the whole body and half of the manubrium of the sternum (specimen size 15 × 5 × 5 cm) was performed with forced ligation of both mammary arteries as a lifesaving surgery. However, definitive perioperative haemostasis was technically challenging, continuous bleeding from wound was still evident.

The planned cardiac surgery was stopped because of a high procedure risk and the recurrence of distant RCC. After refilling the blood volume, the patient was disconnected from the extracorporeal circulation and haemostyptics were administered. However, he was stable only temporarily and there was still need for catecholamine support. Heart failure occurred because of high blood loss (3000 ml) together with unsolved serious aortic stenosis. We tried to eliminate subsequent circulatory collapse with direct heart massage. However, it was ineffective, and the patient died in the operating theatre.

**Fig. 2:**
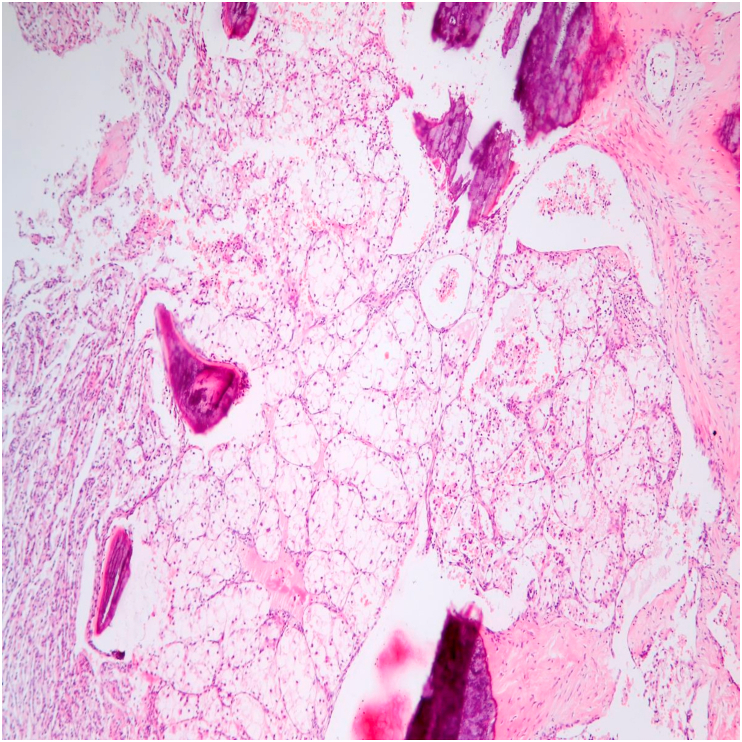
Sternal metastasis of clear cell renal cell carcinoma showing bone destruction (hematoxylin-eosin, original magnification 100x).

During autopsy, there was no locoregional recurrence of RCC and the sternum with adjacent soft tissues was the only distant metastasis found (Fig. 2).

## Discussion

Distant metastases are detected in up to 50% of RCC patients, with bone metastases representing 30–40% cases. Therefore, the first and the only clinical sign of RCC is a pathologic bone fracture in a significant subset of patients.^3^ Unfortunately, the localization of metastasis in our case prevented clinical manifestation and early diagnosis by routinely used imaging methods.

Solitary metastasis of RCC is rare, occurring in less than 5% of patients. Its successful surgical removal significantly improves prognosis and treatment could be curative.^4^ In our case, the patient's performance status (ECOG/WHO score 2) and severe bleeding from the unrecognized metastasis prevented successful completion of the procedure.

Because of the highly vascularised stroma of tumour and the poor quality of newly formed blood vessels, perioperative excessive bleeding during resection is rather common. It is advantageous to use angiographic obliteration in the case of vascularised bone metastasis for prevention of serious bleeding.^5^ We were not able to perform angiographic obliteration before the procedure, because the metastasis was diagnosed only during the procedure. The patient's condition in the operating theatre did not allow his transport to angiographic obliteration.

## Conclusion

We present a serious complication with fatal consequences for the patient, who was regularly followed-up after nephrectomy for RCC. For five years, there were no signs of recurrence. However, RCC is well known for its high tendency for recurrence even after a prolonged period. Preoperative testing (including chest physical examination and x-ray) did not find the solitary metastasis in the sternum. Based on this experience, we recommend more detailed recent restaging (including CT scan of chest and abdomen) before considering cardiac surgery.

## Section headings

Oncology

## Funding

Supported by MH CZ - DRO (UHHK, 00179906), by the programme PROGRES Q40/04 and Q40/11 and by project BBMRI-CZ LM2018125.

## Declaration of competing interest

None declared.
